# A prospective ascertainment of cancer incidence in sub‐Saharan Africa: The case of Kaposi sarcoma

**DOI:** 10.1002/cam4.618

**Published:** 2016-01-28

**Authors:** Aggrey Semeere, Megan Wenger, Naftali Busakhala, Nathan Buziba, Mwebesa Bwana, Winnie Muyindike, Erin Amerson, Toby Maurer, Timothy McCalmont, Philip LeBoit, Beverly Musick, Constantin Yiannoutsos, Robert Lukande, Barbara Castelnuovo, Miriam Laker‐Oketta, Andrew Kambugu, David Glidden, Kara Wools‐Kaloustian, Jeffrey Martin

**Affiliations:** ^1^ Infectious Diseases InstituteMakerere UniversityKampalaUganda; ^2^ University of CaliforniaSan FranciscoCAUSA; ^3^ Moi UniversityEldoretKenya; ^4^ Mbarara University of Science and TechnologyMbararaUganda; ^5^ Indiana UniversityIndianapolisIndiana; ^6^ Makerere University College of Health SciencesKampalaUganda

**Keywords:** Africa, antiretroviral therapy, HIV/AIDS, incidence, Kaposi sarcoma

## Abstract

In resource‐limited areas, such as sub‐Saharan Africa, problems in accurate cancer case ascertainment and enumeration of the at‐risk population make it difficult to estimate cancer incidence. We took advantage of a large well‐enumerated healthcare system to estimate the incidence of Kaposi sarcoma (KS), a cancer which has become prominent in the HIV era and whose incidence may be changing with the rollout of antiretroviral therapy (ART). To achieve this, we evaluated HIV‐infected adults receiving care between 2007 and 2012 at any of three medical centers in Kenya and Uganda that participate in the East Africa International Epidemiologic Databases to Evaluate AIDS (IeDEA) Consortium. Through IeDEA, clinicians received training in KS recognition and biopsy equipment. We found that the overall prevalence of KS among 102,945 HIV‐infected adults upon clinic enrollment was 1.4%; it declined over time at the largest site. Among 140,552 patients followed for 319,632 person‐years, the age‐standardized incidence rate was 334/100,000 person‐years (95% CI: 314–354/100,000 person‐years). Incidence decreased over time and was lower in women, persons on ART, and those with higher CD4 counts. The incidence rate among patients on ART with a CD4 count >350 cells/mm^3^ was 32/100,000 person‐years (95% CI: 14–70/100,000 person‐years). Despite reductions over time coincident with the expansion of ART, KS incidence among HIV‐infected adults in East Africa equals or exceeds the most common cancers in resource‐replete settings. In resource‐limited settings, strategic efforts to improve cancer diagnosis in combination with already well‐enumerated at‐risk denominators can make healthcare systems attractive platforms for estimating cancer incidence.

Cancer incidence is one of the most fundamental parameters in cancer epidemiology. Incidence encompasses both the natural history of a malignancy and the effects of interventions to reduce occurrence 1. Accurate estimates of cancer incidence are vital elements in ascertaining the etiology of cancers, planning for public health burden, and monitoring the effects of interventions. In resource‐rich settings, given the better equipped medical infrastructure, virtually all instances of cancer are diagnosed and recorded. These diagnoses are then placed into context of the underlying denominator of persons at risk by the creation of incidence rates 2. The denominators are typically derived from municipally funded complete enumerations (i.e., a census) of geographic populations. In contrast, in resource‐limited settings, such as sub‐Saharan Africa, there is limited infrastructure for cancer diagnosis, and even when diagnosed, not all cancers are formally recorded 3. Further, there are challenges in enumerating the denominator from which cancers arise. The WHO‐sponsored Cancer Incidence in Five (“CI5”) Continents project deemed only 4 out of 25 registries from countries in sub‐Saharan Africa to have sufficient quality 4, 5, and even within these countries, there are issues in both ascertainment of total cancer cases and the underlying denominator.

Kaposi sarcoma (KS) is an example of a malignancy in a resource‐limited setting which would benefit from knowledge about incidence. From a perspective of percentage of all recorded cancers, KS was among the most common cancers in sub‐Saharan Africa even before the human immunodeficiency virus (HIV) epidemic 6, 7, and it experienced explosive growth as HIV infection spread 8, 9. The clinical relevance of KS includes both cosmetic disfigurement and considerable morbidity and mortality. In persons untreated for HIV, 1‐year mortality after KS diagnosis in sub‐Saharan Africa is 60% to 70% 10, 11. Even among persons treated with antiretroviral therapy (ART), those with KS have about a fourfold higher rate of death 12. In resource‐rich settings, ART has substantially reduced KS incidence, but because of the lack of robust sources of incidence data, the status in sub‐Saharan Africa is less clear aside from an initial report from South Africa 13. As is true for many cancers, changes in KS incidence in resource‐replete settings cannot necessarily be extrapolated to resource‐limited ones. Differences between settings regarding the strain of the etiologic viral agent (Kaposi sarcoma‐associated herpesvirus, KSHV), ambient HIV strains, human host, and potentially other environmental cofactors dictate that KS incidence must be directly measured in Africa for it to be relevant.

To overcome the challenges inherent in a resource‐limited setting, we used a newly assembled collection of healthcare system‐derived databases, the International Epidemiological Databases to Evaluate AIDS (IeDEA) Consortium in East Africa, to derive a well‐substantiated (in terms of numerator and denominator) estimate of cancer incidence in a large representative population of HIV‐infected adults in sub‐Saharan Africa. We focused on KS, not only because of its ease of measurement and clinical relevance, but also to demonstrate how adding the selective measurements to an already well‐enumerated healthcare system‐based population has the potential to be a powerful platform for the study of other cancers.

## Methods

### Overall design

We performed a cohort analysis among HIV‐infected adults (≥18 years old) receiving care at one of three medical centers in Kenya and Uganda that participate in the East Africa IeDEA Consortium. Established in 2005 by the U.S. National Institutes of Health, the IeDEA Consortium has as its main procedural objective the harmonizing of data collected by geographically disparate, but representative, cohorts of persons infected with HIV or at risk for HIV 14, 15, 16. The scientific goal is to generate inferences about the natural or treated history of HIV, particularly regarding uncommon exposures or outcomes for which large samples are needed.

### Study population

We included data from HIV‐infected adults who were receiving care at one of three medical care systems: the Immune Suppression Syndrome (ISS) Clinic located at the Mbarara Regional Referral Hospital in Mbarara, Uganda 17; the Infectious Diseases Institute (IDI) at Mulago National Referral Hospital in Kampala, Uganda 18; and the Academic Model Providing Access to Healthcare (AMPATH) network in western Kenya 19. Briefly, the ISS Clinic, located in southwestern Uganda, has cared for over 20,000 HIV‐infected adults since it began in 1998. Since 2002, the IDI has offered HIV/AIDS care to over 30,000 patients; the patient base is mainly from the greater urban Kampala area with a small proportion from outside Kampala. Of note, during 2008 to 2011, the IDI conducted a treatment study of KS, in which all patients became part of the IDI clinic. AMPATH was established in 2001 and has cumulatively enrolled over 160,000 HIV‐infected patients at 60 different clinics. The average number of patients cared for per year during the study period was 55,502 at AMPATH, 10,428 at IDI, and 8329 at the ISS Clinic. Each of these systems provides the standard‐of‐care management of HIV disease, including counseling, free cotrimoxazole prophylaxis, and free ART. We included any patient with at least one clinic visit between January 2007 and July 2012, incorporating all patients newly enrolling in January 2007 or later as well as those who enrolled prior to 2007. Patients were followed, for the purpose of this analysis, until the earliest occurrence of KS, transfer to another healthcare facility, death, or administrative database closure. Approval for this research was granted by each site's institutional review board.

### Measurements

#### KS

Prior to 2007, at all sites, the diagnosis of KS was largely clinical only (i.e., by visual inspection) with rare pathologic confirmation. In January 2007, skin punch biopsy capacity for histopathologic diagnosis (provided free to patients) was introduced at IDI, followed in October 2008 at the ISS Clinic and AMPATH. Through support from IeDEA, clinicians received training in the recognition of KS, the importance of early pathologic diagnosis, and how to perform a biopsy. Biopsy interpretations were adjudicated by dermatopathologists at the University of California, San Francisco, who had available to them, at their discretion, the use of immunohistochemical staining against the latency‐associated nuclear antigen of KSHV. For the purpose of this analysis, a KS diagnosis was defined by the presence of a clinical‐only diagnosis of KS or a clinical KS diagnosis accompanied by definitive or indeterminate pathologic confirmation. When there were both a date of diagnosis on clinical grounds and a date of pathologic confirmation, we used the earlier date as the overall date of diagnosis. Patients with a clinical diagnosis and a negative biopsy were not considered to have KS.

#### Other variables

Age, sex, weight, ART use, hemoglobin, and CD4+ T‐cell count were obtained from the ambient electronic medical record databases. Because ART adherence information was not recorded routinely, we assumed that once started on ART, patients continued on ART.

### Statistical analysis

In an analysis of KS prevalence, we first determined the frequency of KS among patients upon enrollment in their respective medical systems, limiting to those newly enrolled on or after 1 January 2007 who had also not yet started ART. Prevalence upon clinic enrollment in ART‐unexposed patients serves as a rough correlate of KS incidence in the community and is one measure of the burden of KS facing HIV care clinics. Because KS might go unnoticed at an administratively demanding initial clinic visit, we assumed that any KS recorded within 30 days of enrollment was present at enrollment and was therefore prevalent.

For the incidence analysis, we studied all patients who had no diagnosis of KS, as of the time they became eligible for the analysis, for the subsequent occurrence of KS. Therefore, 1 January 1 2007 was considered time zero for patients enrolled prior to then, and the date of the initial clinic visit was time zero for those enrolled after 1 January 2007. We calculated incidence rates, as total new cases of KS divided by the at‐risk person‐time (expressed per 100,000 person‐years), for the overall population as well as subgroups defined by age, sex, calendar year, medical center, ART status, and CD4+ T‐cell count. For ART status, we classified the population according to person‐time in which ART was not used (“non‐ART use”) and person‐time in which ART was used (“ART use”). Given the importance of capturing the early time period after ART initiation 20, we also evaluated person‐time in which ART was used among patients who initiated ART under our observation (“new ART use”). As such, “new ART use” is a subset of the “ART use” person‐time. Age‐standardized incidence rates (ASIR) were estimated using both the new 21 and old 22, 23 WHO world standard population. Because we only had direct data on persons 18 years and older, we limited our primary ASIR estimation to adults. In an additional analysis, we estimated ASIR for an entire population age range by assuming zero incidence of KS among those <15 years. Because we recognize that zero incidence is unlikely, we consider this ASIR to be a “best case,” that is, a minimum estimate. In a sensitivity analysis, we assumed that any KS occurring within 30 days following ART initiation was present but unrecognized at the start of ART, and hence, we reset diagnosis date to the day prior to ART. Change over time in either prevalence or incidence was assessed with score tests for trend 24. All analyses were performed with Stata (version 13.1, Stata Corp., College Station, TX, USA).

## Results

### Characteristics of the study populations

**Figure 1:**
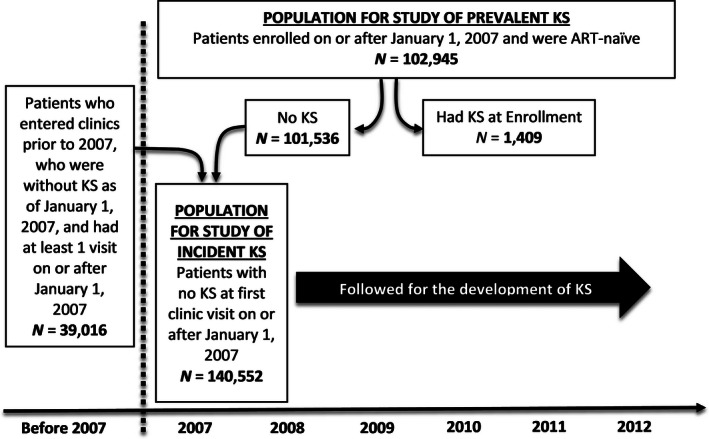
Summary of the process of patient selection for the study of Kaposi sarcoma (KS) prevalence and incidence in the three healthcare systems in East Africa. ART denotes antiretroviral therapy.

In the analysis of prevalent KS upon medical center enrollment, we evaluated 102,945 HIV‐infected adults (See Figure 1), 10,519 (10%) from the ISS–Uganda, 7332 (7%) from the IDI–Uganda, and 85,094 (83%) from the AMPATH–Kenya. Their median age was 34 years, women comprised 66% of the sample, and the median CD4+ T‐cell count was 253 cells/mm^3^ (Table 1). In the incidence analysis, we followed 140,552 HIV‐infected adults (See Figure 1), 15,437 (11%) from the ISS–Uganda, 16,493 (12%) from the IDI–Uganda, and 108,622 (77%) from the AMPATH–Kenya (Table 2). At time zero, their median age was 35 years, women comprised 67% of the sample, 16% were on ART, and the median CD4+ T‐cell count was 270 cells/mm^3^. Of the 117,764 patients not on ART at time zero, 75,122 subsequently started ART during follow‐up with 9874 of these initiating ART at the enrollment visit.

**Table 1:** Characteristics of antiretroviral therapy‐naive HIV‐infected adults upon enrollment in one of three healthcare systems in East Africa (Prevalent KS study population)

Characteristic	ISS *n* = 10,519	IDI *n* = 7,332	AMPATH *n* = 85,094	Overall *n* = 102,945
Age, years^a^	31 (26–38)^b^	33 (27–40)	35 (28–43)	34 (28–42)
Age category, years				
18–19	2.9%	2.5%	1.5%	1.7%
20–24	16%	13%	10%	11%
25–29	23%	18%	17%	18%
30–34	21%	20%	20%	20%
35–39	16%	19%	18%	17%
40–44	10%	13%	13%	13%
45–49	5.8%	7.5%	9.3%	8.8%
50–54	3.0%	3.4%	5.7%	5.3%
55–59	1.3%	1.8%	3.2%	2.9%
60–64	0.8%	0.9%	1.6%	1.5%
65–69	0.5%	0.5%	0.6%	0.6%
70–74	0.2%	0.3%	0.3%	0.3%
75–79	0.1%	0.05%	0.1%	0.1%
≥ 80	0.02%	0.01%	0.04%	0.04%
Male sex	37%	35%	33%	34%
Weight, Kg^c^	54 (48–61)^b^	55 (49–62)	55 (49–62)	55 (49–62)
CD4+ T‐cells, cells/mm^3,^ d	296 (136–493)^b^	270 (105–484)	247 (102–439)	253 (105–447)
CD4+ T‐cells category, cells/mm^3^				
0–50	12%	15%	14%	14%
51–100	8.1%	9.0%	10%	10%
101–200	16%	15%	18%	18%
201–350	23%	22%	22%	22%
351–500	18%	15%	16%	16%
≥ 500	24%	24%	19%	20%
Hemoglobin, mg/dl^e^	13 (11–14)^b^	12 (11–14)	12 (10–13)	12 (10–14)

ISS denotes Immune Suppression Syndrome Clinic in Mbarara, Uganda; IDI denotes Infectious Diseases Institute in Kampala, Uganda; AMPATH denotes Academic Model for Providing Access to Healthcare in western Kenya.

a 0.2% missing age.

b Median (interquartile range) unless otherwise noted.

c 2.1% missing weight.

d 16% missing CD4 count.

e 20% missing hemoglobin.

**Table 2:** Characteristics at the beginning of their respective period of observation among all HIV‐infected adults who were followed for the development of incident Kaposi sarcoma at one of three healthcare systems in East Africa (Incident KS study population)

Characteristic	ISS *n* = 15,437	IDI *n* = 16,493	AMPATH *n* = 108,622	Overall *n* = 140,552
Age, years^a^	33 (27–40)^b^	35 (29–41)	35 (29–43)	35 (29–43)
Age category, years				
18–19	2.1%	1.2%	1.2%	1.3%
20–24	13%	9.2%	9.8%	10%
25–29	20%	16%	16%	17%
30–34	19%	20%	19%	19%
35–39	18%	21%	18%	19%
40–44	13%	17%	14%	14%
45–49	7.0%	8.0%	9.5%	9.0%
50–54	3.4%	3.7%	5.5%	5.1%
55–59	1.9%	2.1%	3.2%	2.9%
60–64	1.0%	1.1%	1.6%	1.4%
65–69	0.5%	0.5%	0.6%	0.6%
70–74	0.2%	0.2%	0.3%	0.3%
75–79	0.1%	0.07%	0.1%	0.1%
≥ 80	0.03%	0.03%	0.04%	0.04%
Male sex	37%	33%	32%	33%
Weight, Kg^c^	56 (49–63)	57 (51–64)	56 (50–63)	56 (50–63)
CD4+ T cells, cells/mm^3,^ d	316 (160–510)	292 (159–466)	262 (122–440)	270 (129–449)
CD4+ T‐cells category, cells/mm^3^				
0–50	9.3%	8.9%	12%	11%
51–100	6.9%	6.7%	9.2%	8.7%
101–200	15%	16%	18%	18%
201–350	24%	28%	25%	25%
351–500	19%	18%	17%	17%
≥ 500	26%	22%	19%	20%
Hemoglobin, mg/dl^e^	13 (11–14)	13 (11–14)	12 (10–14)	12 (10–14)
ART in use	20%	26%	14%	16%

ISS denotes Immune Suppression Syndrome Clinic in Mbarara, Uganda; IDI denotes Infectious Diseases Institute in Kampala, Uganda; AMPATH denotes Academic Model for Providing Access to Healthcare in western Kenya.

a 0.4% missing age.

b median (interquartile range) unless otherwise noted.

c 3.7% missing weight.

d 18% missing CD4 count.

e 28% missing hemoglobin.

### Prevalence of KS at clinic enrollment

**Figure 2:**
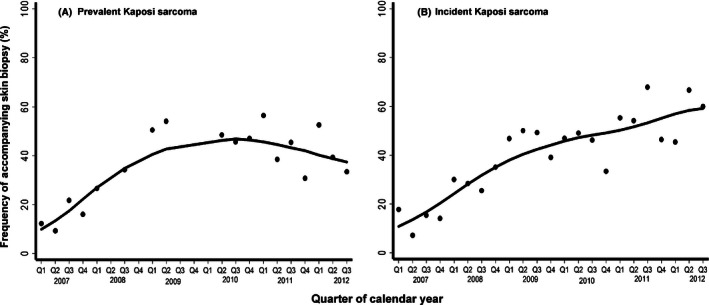
Frequency of accompanying skin biopsy at the time of diagnosis of Kaposi sarcoma (KS) among HIV‐infected patients over time at three healthcare systems in East Africa. Panel A shows instances of prevalent KS diagnosis, and panel B shows cases of incident KS. Line represents locally weighted scatterplot smoothing (LOWESS). Q represents quarter of the calendar year.

**Figure 3:**
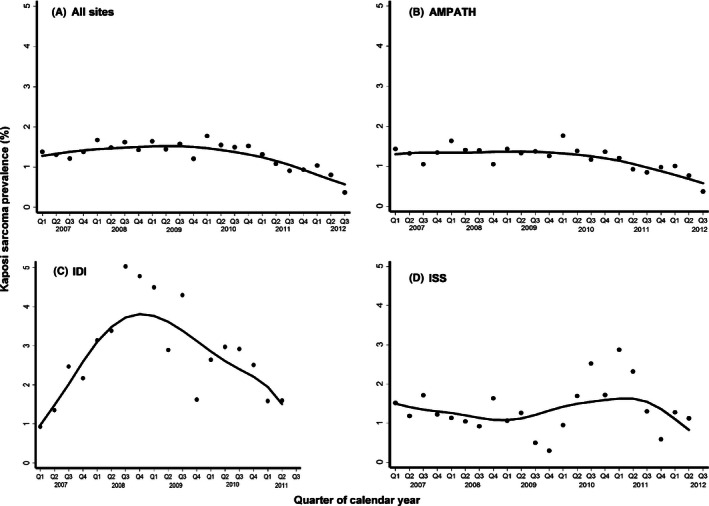
Prevalence of Kaposi sarcoma at the time of clinic enrollment among HIV‐infected patients over time at three healthcare systems in East Africa. Line represents locally weighted scatterplot smoothing (LOWESS). AMPATH denotes Academic Model Providing Access to Healthcare in western Kenya; ISS denotes Immune Suppression Syndrome Clinic in Mbarara, Uganda; and IDI denotes Infectious Diseases Institute in Kampala, Uganda. Q represents quarter of the calendar year.

Among 102,945 patients, a total of 1409 KS diagnoses were made at clinic enrollment, of which 63% were clinical only, 36% were biopsy confirmed, and 0.9% were clinical with an indeterminate biopsy. Over time, the frequency of diagnoses accompanied by biopsy increased (to about 50%) and then plateaued (Fig. 2; Panel A). Overall, the prevalence of KS at clinic enrollment was 1.4% (95% confidence interval (CI): 1.3–1.4); it was 1.3% at the ISS–Uganda, 2.8% at the IDI–Uganda, and 1.3% at the AMPATH–Kenya (Fig. 3). At the IDI–Uganda, the spike in prevalence between 2008 and 2010 likely reflects the enrichment of patients with KS from the aforementioned treatment trial. Thus, KS prevalences at the AMPATH–Kenya, and at the ISS–Uganda, are better representatives of disease burden in their underlying communities. At both of these sites, prevalence was slightly above 1% at the beginning of 2007. Prevalence fell at the AMPATH–Kenya, over time (*P* for trend <0.001), but, at the ISS–Uganda, there was no evidence for systematic change in prevalence (*P* for trend = 0.52).

### Incidence of KS

Among the 140,552 patients who began observation without KS, we subsequently followed them for incident KS over 319,605 person‐years (median 1.8 years; interquartile range (IQR) 0.4–4.2 years). Among non‐ART users, the total person‐time was 88,566 person‐years (median 1.9 years; IQR 0.4–4.2 years), while among ART users, it was 231,038 person‐years (median 1.8 years; IQR 0.5–3.5 years) (135,523 person‐years among new ART users; median 1.3 years; IQR 0.4–2.9 years). Observation ended with the development of KS in 1025 patients, death in 8609, transfer to another medical facility in 6214, administrative closure in 76,775, and loss to follow‐up in 47,929.

**Figure 4:**
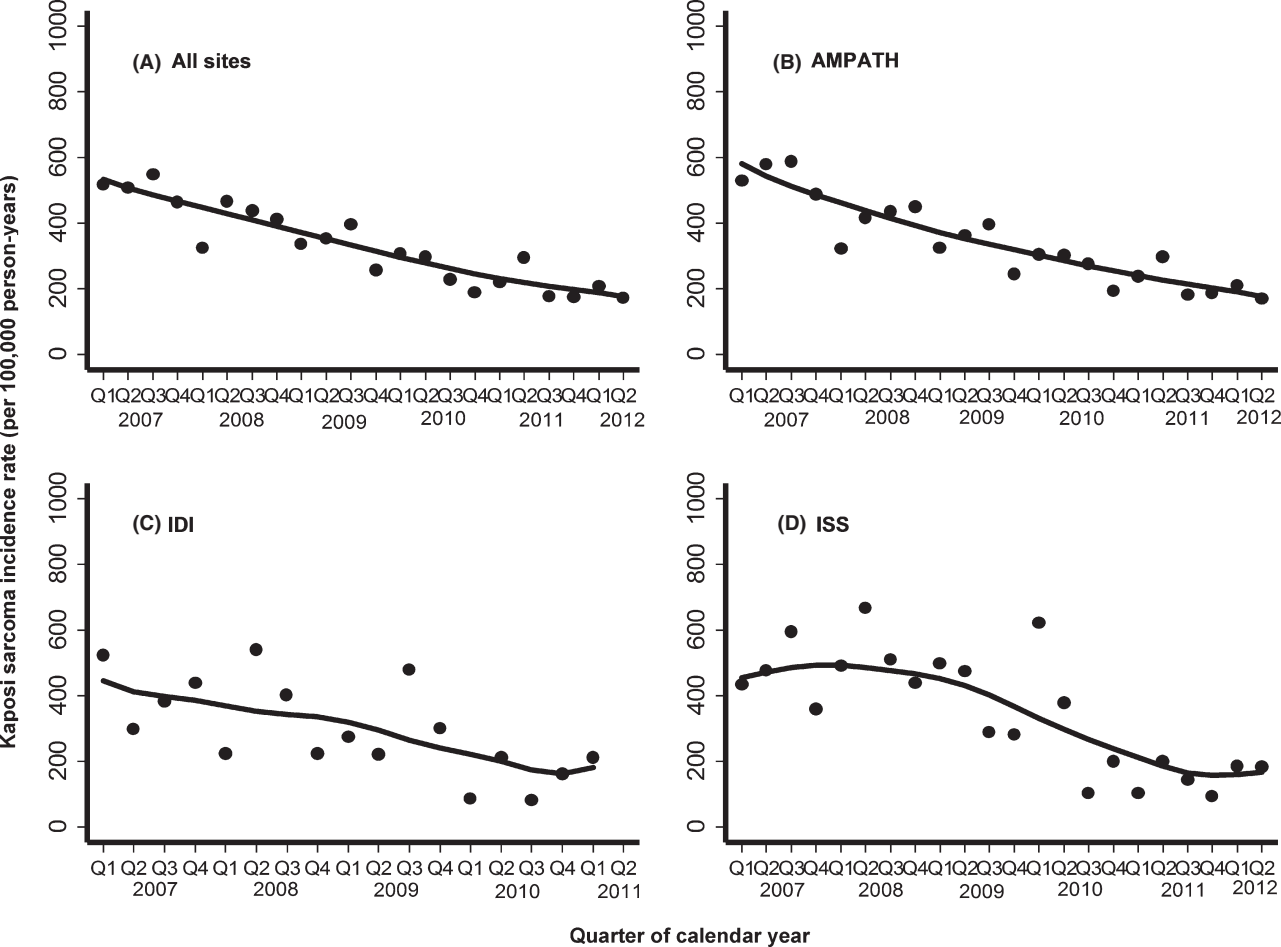
Incidence rate of Kaposi sarcoma (per 100,000 person‐years) over time among HIV‐infected patients at three healthcare systems in East Africa. Line represents locally weighted scatterplot smoothing (LOWESS). AMPATH denotes Academic Model Providing Access to Healthcare in western Kenya; ISS denotes Immune Suppression Syndrome Clinic in Mbarara, Uganda; and IDI denotes Infectious Diseases Institute in Kampala, Uganda. Q represents quarter of the calendar year.

Of the 1025 incident KS diagnoses (76% at the AMPATH–Kenya and 12% at both the ISS–Uganda, and IDI–Uganda), 63% were clinical only, 36% were biopsy confirmed, and 1.1% were biopsy indeterminate. Over time, the frequency of diagnoses accompanied by biopsy steadily increased (Fig. 2; Panel B). The overall KS incidence rate was 321/100,000 person‐years (95% CI: 302–341/100,000 person‐years), which did not substantively differ across sites: 321/100,000 person‐years at the AMPATH–Kenya, 337/100,000 person‐years at the ISS–Uganda, and 301/100,000 person‐years at the IDI—Uganda (Table 3). When limiting inference to our observed population of adults age ≥18 years, the ASIR was 334/100,000 person‐years (95% CI: 314–354/100,000 person‐years) using the new WHO standard population and was 340/100,000 (95% CI: 319–361/100,000 person‐years) using the older WHO standard population. Extending inference to the entire population age range (including children), the ASIR was 247/100,000 person‐years in the best‐case scenario, assuming zero incidence among children. Over time, overall incidence decreased, from a peak in 2007 of 509/100,000 person‐years to 222/100,000 person‐years in 2012 (*P* for trend <0.001; Fig. 4). The incidence of KS generally reduced over calendar time similarly across sites.

**Table 3:** Incidence rate of Kaposi sarcoma among HIV‐infected adults at three healthcare systems in East Africa

Group	All Patients			Non‐ART Users			ART Users			New ART Users		
	Female *n* = 94,334	Male *n* = 46,218	Overall *n* = 140,552	Female *n* = 84,552	Male *n* = 43,039	Overall *n* = 127,591	Female *n* = 66,470	Male *n* = 31,440	Overall *n* = 97,910	Female *n* = 51,283	Male *n* = 23,839	Overall *n* = 75,122
Age, Years												
18–19	479^a^(249,920)	921(230,3681)	524(290,947)	877(418,1840)	1300(183,9229)	914(457,1828)	185(46,739)	713(100,5100)	245(79,760)	201(50,804)	788(111,5591)	268(86,829)
20–24	248(190,323)	1080(746,1565)	336(271,417)	340(231,499)	476(214,1059)	359(254,508)	198(137,287)	1700(1100,2500)	323(245,426)	214(141,325)	1882(1185,2986)	356(261,485)
25–29	233(190,286)	789(622,1001)	333(285,388)	206(139,305)	939(660,1335)	363(279,471)	245(193,312)	697(505,961)	319(263,386)	286(218,377)	945(675,1323)	397(321,491)
30–34	239(198,288)	711(590,856)	358(314,409)	214(147,312)	1025(777,1353)	440(352,550)	248(200,308)	568(442,730)	326(277,384)	319(251,406)	731(557,960)	424(354,508)
35–39	258(214,311)	493(408,596)	337(295,385)	406(303,544)	772(568,1048)	525(425,648)	206(161,262)	403(316,512)	273(230,324)	318(243,415)	635(491,822)	429(356,516)
40–44	178(138,230)	440(358,540)	280(238,289)	189(116,308)	584(398,858)	325(240,440)	174(129,236)	400(314,510)	265(220,321)	253(178,360)	609(458,811)	391(313,488)
45–49	157(108,227)	387(293,510)	253(203,316)	120(54,267)	663(418,1052)	311(209,464)	171(112,259)	313(222,443)	234(179,305)	243(153,385)	488(335,712)	348(259,465)
50–54	259(172,390)	419(293,510)	330(252,433)	278(132,583)	449(214,942)	343(203,579)	252(154,411)	410(273,617)	326(238,446)	367(217,619)	492(306,791)	426(300,606)
55–59	269(160,455)	391(246,620)	326(231,462)	435(195,969)	795(398,1590)	587(348,991)	210(105,419)	278(149,516)	243(153,385)	230(96,553)	395(198,791)	310(180,533)
60–64	90(22,359)	456(245,847)	271(154,478)	168(24,1190)	854(321,2276)	470(195,1128)	61(8.6,436)	347(156,773)	209(99,437)	112(16,793)	637(286,1418)	381(182,799)
65–69	333(83,1322)	214(54,857)	261(98,695)	1071(268,4283)	846(211,3381)	945(355,2518)	0^b^(0,900)	0^b^(0,527)	0^b^(0,332)	0^b^(0,1317)	0^b^(0,878)	0^b^(0,527)
70–74	807(202,3225)	452(113,1805)	579(217,1543)	0^b^(0,4099)	761(107,5401)	459(65,3256)	1200(310,5000)	321(45,2300)	634(205,1967)	835(118,5925)	0^b^(0,1677)	298(42,2113)
Age‐standardized rates												
New WHO^c^	249(227,271)	602(550,654)	334(314,354)	347(295,400)	771(663,879)	471(423,519)	215(192,238)	579(516,642)	270(249,291)	255(224,286)	725(638,812)	340(311,369)
Old WHO^d^	254(232,276)	616(563,669)	340(319,361)	361(306,416)	786(676,896)	483(433,533)	214(191,237)	593(528,658)	271(250,292)	254(223,285)	741(652,830)	341(312,370)
New best case^e^	184(168,200)	445(407,483)	247(232,262)	257(218,296)	570(490,650)	348(312,384)	159(142,176)	428(381,475)	199(184,214)	189(166,212)	535(471,599)	251(230,272)
CD4+ T cell, cells/mm^3^ f												
0–50	1392(749,2587)	1938(1073,3500)	1633(1065,2505)	1379(620,3070)	2387(1194,4774)	1818(1077,3069)	1400(530,3800)	1300(416,4000)	1357(647,2847)	1696(547,5260)	1304(326,5213)	1500(630,3600)
51–100	728(347,1526)	494(185,1316)	621(344,1121)	1576(708,3508)	1061(342,3289)	1357(706,2607)	172(24,1200)	190(27,1300)	180(45,721)	0^b^(0,946)	0^b^(0,1025)	0^b^(0,492)
101–200	181(86,380)	334(180,621)	248(154,399)	177(44,709)	754(314,1811)	391(186,820)	183(76,439)	215(89,516)	197(106,367)	108(27,433)	340(141,816)	211(101,442)
201–350	125(73,216)	325(210,504)	200(142,281)	157(65,376)	940(557,1587)	406(259,636)	111(56,223)	129(58,286)	118(70,200)	156(74,327)	192(80,462)	169(96,298)
351–500	74(35,155)	48(12,192)	66(34,127)	135(51,359)	164(41,655)	143(64,319)	46(15,144)	0^b^(0,125)	32(10,99)	81(26,253)	0^b^(0,246)	58(19,179)
>350	66(39,112)	80(36,179)	70(45,109)	141(76,263)	161(60,428)	146(87,247)	29(11,76)	40(10,161)	32(14,70)	40(13,125)	43(6.0,302)	41(15,109)
>500	60(29,126)	121(46,323)	74(41,133)	146(66,325)	158(39,630)	149(74,298)	13(1.9,95)	99(25,394)	31(10,98)	0^b^(0,98)	118(17,834)	22(3.1,154)
Study Site												
ISS	234(181,304)	534(421,677)	337(283,402)	263(173,399)	770(520,1139)	404(304,538)	220(158,306)	453(336,611)	307(246,383)	294(199,436)	702(499,988)	440(340,569)
IDI	222(173,286)	474(367,612)	301(252,360)	220(148,329)	563(374,847)	314(236,418)	224(162,309)	431(311,597)	293(233,369)	411(278,608)	959(657,1398)	584(445,767)
AMPATH	229(207,253)	534(484,589)	321(300,345)	280(234,334)	805(686,946)	436(387,491)	211(187,238)	444(391,503)	282(259,308)	272(238,311)	612(534,701)	374(340,411)
All Patients	229(210,250)	526(483,574)	321(302,341)	267(230,311)	762(663,877)	411(371,455)	213(192,237)	443(398,494)	286(265,309)	283(251,319)	645(573,728)	394(362,429)

a Incidence rate per 100,000 person‐years with 95% confidence interval.

b No cases were observed despite observing person‐time.

c Uses most recent WHO world standard population (2000–2025) 21; limited to age 15 and older, younger age groups are excluded.

d Uses older WHO standard population 22, 23; limited to age 15 and older, younger age groups are excluded. This standard is used in the Cancer in Five Continents report 4.

e Uses most recent WHO world standard population (2000–2025) 21. Includes all age groups but assumes zero incidence of KS among children aged <15 years. This is therefore considered a best‐case scenario or minimum estimate.

f CD4 categories represent time‐updated values.

When evaluating KS incidence among subgroups, there was no clear pattern associated with age, but incidence was higher among men at 526/100,000 person‐years (95% CI: 483–574/100,000 person‐years) compared to women (229/100,000 person‐years; 95% CI: 210–250/100,000 person‐years), and higher with decreasing CD4 count (Table 3). Among non‐ART users, incidence was 411/100,000 person‐years (95% CI: 371–455/100,000 person‐years), compared to 286/100,000 person‐years (95% CI: 265–309/100,000 person‐years) among all ART users and 394/100,000 person‐years (95% CI: 362–429/100,000 person‐years) when limited to the new ART users. The differences between ART users and non‐ART users became more apparent when stratified by CD4+ T‐cell count. For example, for patients with CD4+ T‐cell counts above 350 cell/mm^3^ (the commonly perceived immunologic safe haven), KS incidence was 146/100,000 person‐years (95% CI: 87–247/100,000 person‐years) among non‐ART users, 32/100,000 person‐years (95% CI: 14–70/100,000 person‐years) among all ART users, and 41/100,000 person‐years (95% CI: 15–109/100,000 person‐years) among new ART users.

In the sensitivity analysis, we found 94 patients diagnosed with KS within the first 30 days of initiating ART, and for these patients, we reset the date of KS diagnosis to the day prior to ART start. The resulting revised incidence rate was 516/100,000 person‐years (95% CI: 471–565/100,000 person‐years) among those not on ART, 245/100,000 person‐years (95% CI: 226–266/100,000 person‐years) among all ART users, and 325/100,000 person‐years (95% CI: 296–357/100,000 person‐years) among only new ART users.

## Discussion

When estimating cancer incidence, resource‐limited settings, in contrast to resource‐rich ones, have difficulties in both the ascertainment of cancer cases (i.e., the numerator in the incidence calculation) and the true at‐risk population (i.e., the denominator). We have demonstrated how a large healthcare system‐based platform–East Africa IeDEA–can overcome many of the prior limitations of estimating cancer incidence in sub‐Saharan Africa and be used to precisely estimate the incidence of KS, one of the most common cancers in the region. The cornerstone of this approach is the very large population that is enumerated longitudinally at the individual person level in electronic databases with standardized coding at the IeDEA sites. To this large population, we enhanced KS diagnosis through clinician training and provision of skin punch biopsy equipment for histopathologic diagnosis. These strategic efforts increased the specificity of diagnosis compared to the heretofore convention of clinical‐only diagnosis 25; they conceivably may have also increased sensitivity. Accordingly, our estimate of KS incidence is among the first prospective estimates of cancer incidence at the individual level in East Africa and, we believe, among the least biased and most precise.

Conventionally, the gold standard approach for estimating cancer incidence in both resource‐rich and resource‐limited settings is to derive it within a defined geographic area. In resource‐rich settings, this is accomplished by combining data from the population‐based cancer registries (numerator) and a relevant geographic population census (denominator). The resultant incidence estimates are both accurate and precise because functional underlying healthcare systems diagnose and record most, if not all, cancers that occur, cancer registries collect these diagnoses, and up‐to‐date census data from large geographic regions enumerate the at‐risk population. In contrast, in sub‐Saharan Africa, the CI5 project deemed that only four cancer registries–each covering only a small region of a single country–had data of sufficient quality 4 (Table 4). Furthermore, even within these four registries, there are several challenges that threaten the veracity of the incidence estimates, particularly for KS. First, cancer case ascertainment is largely derived from the most predictably efficient sources: pathology records, oncology clinics, and inpatient wards. Yet, cancer in resource‐limited settings is frequently diagnosed on clinical grounds alone in primary care settings, may never get cared for in any higher facility, and hence never gets systematically recognized. This is especially true for KS, and it is unclear (but unlikely) that any of the four CI5‐worthy registries scoured all of the outpatient records of the HIV care clinics in their region to detect all clinical KS diagnoses. In the registry believed to have the most comprehensive coverage of care venues (Blantyre, Malawi), only 17% of KS cases were pathologically confirmed 26, and this percentage is nonetheless still likely an overestimate unless all HIV primary care clinics were evaluated. Second, lack of resources threatens the frequency and accuracy of the underlying census data that inform the incidence denominator. In Uganda, for example, the most recent census was postponed for two years 27. In addition, with patients from rural areas coming to urban areas for health care when they are ill, it is unclear what the effective population base for the urban‐based registries is. Finally, in both resource‐limited and resource‐rich settings, the geographic‐based approach of incidence estimation typically leaves us not knowing which KS cases are HIV infected, and the denominator of the at‐risk HIV‐infected persons is also not precisely known. Thus, we cannot directly learn the incidence of KS in the group that is most affected–those with HIV infection.

**Table 4:** Estimates of Kaposi sarcoma (KS) incidence rates in sub‐Saharan Africa

Authors, year of publication	Period of study	Setting; Region	No. of persons; total person‐time^a^	Method of KS diagnosis	Method of at‐risk person‐time ascertainment	Incidence rate of Kaposi sarcoma (95% CI)^b^				Comment
						All persons^c^	All HIV‐infected adults	HIV‐infected adults on ART	HIV‐infected adults off ART	
Mbulaiteye et al. 8	1998–2002	1 HIV clinic; Kyadondo County, Uganda	12,607; 21,667	Matched cancer registry to clinic database	Clinic database; from enrollment to database closure unless known death				240 (183,321)	Unknown to what extent clinical‐only KS diagnoses included Person‐time likely overestimated Matching process imperfect; lacked unique IDs
Asiimwe et al. 29	2003–2008	Home‐based HIV treatment program; Tororo, Uganda	1121; 5294	Pathologic	Clinical trial database			340 (201,537)		KS was one of the outcomes of this clinical trial
Bohlius et al. 13	2004–2010	3 HIV clinics; Cape Town and Johannesburg, South Africa	18,254; 37,488	Not stated	Clinic database		432 (368,504)	138 (102,187)	1682 (1406,2011)	Historically, pathologic confirmation of KS not common in area, and clinical diagnoses may lack specificity.
Rohner et al. 30	2004–2010	6 HIV clinics: Botswana, South Africa, Zambia, Zimbabwe	159,994; 316,784	“often only clinically diagnosed”	Clinic databases			173^d^ (159,188)		Clinical diagnoses may lack specificity
Akarolo‐Anthony et al. 28	2009–2012	2 HIV clinics: Abuja, Nigeria	17,826; 163,265	Matched cancer registry to clinic database	Clinic database; from enrollment to database closure		4.9 (2.1, 9.7)			Unknown to what extent clinical‐only KS diagnoses included Matching process imperfect; lacked unique IDs
Cancer in 5 Continents –Vol. X 4	2003–2007	General population; Kyadondo County, Uganda	M: 889,476F: 979,862	Predominantly pathologic	Local census	M: 19.3F: 15.3				Mostly pathologically confirmed KS. Unclear if clinical diagnoses from HIV primary care clinics were included Uses census data from 2002 with subsequent extrapolation
	2003–2007	General population; Blantyre, Malawi	M: 472,750F: 466,785	Clinical and pathologic	Local census	M: 72.3F: 35.0				Unclear if clinical diagnoses from HIV primary care included Census data from 1998 & 2008 with intercensus interpolation
	2003–2007	General population; Eastern Cape, South Africa	M: 486,176F: 589,499	Clinical and pathologic	Local census	M: 1.7F: 1.1				Unclear if clinical diagnoses from HIV primary care included Census data were available for 2001 with projection to 2005
	2003–2006	General population;Harare, Zimbabwe	M: 717,988F: 712,168	Clinical and pathologic	Local census	M: 28.7F: 18.4				Unclear if clinical diagnoses from HIV primary care included Census data were available for 2002
Current study	2007–2012	62 HIV clinics; western Kenya and Uganda	140,552;321,119	Clinical and pathologic	Clinic database		321 (302,341)	286 (265,309)	411 (371,455)	All clinics received training on KS diagnoses as well as equipment to provide free biopsies

M denotes male; F denotes female.

a In person‐years.

b Per 100,000 person‐years.

c Age‐standardized incidence rate (ASIR).

d Adult population only; incidence was also separately reported in children.

Instead of targeting a geographic population, we used healthcare populations to estimate KS incidence in HIV‐infected individuals, an approach that has been attempted by others in Africa (Table 4). Prior to the advent of potent ART in the country, Mbulaiteye et al. used a record linkage algorithm to match data from an HIV primary care clinic in Kampala, Uganda, to the local cancer registry 8. KS incidence among these untreated HIV‐infected adults was 240/100,000 person‐years. Several reasons likely explain why their estimate is considerably lower than ours. First, they relied upon a pathology department‐based cancer registry during an era when clinical diagnosis alone was quite common. Second, the person‐time denominator was assembled by counting the time from each patient's clinic enrollment to administrative database closure unless a death was definitively known; this likely overestimated the at‐risk person‐time and underestimated KS incidence. Finally, without the ability to match on unique ID number, the record linkage process was admittedly imperfect and may have resulted in failure of some KS diagnoses to be matched. From within the ART era, Asiimwe et al. 28 estimated KS incidence in the context of a home‐based clinical trial of ART monitoring strategies in Uganda. Because of the trial setting, the patients were all presumably followed closely, and all KS diagnoses were biopsy confirmed, thus enhancing the sensitivity and specificity of KS ascertainment. At‐risk person‐time was also carefully measured. Their “on‐ART” KS incidence estimate (340/100,000 person‐years) was higher than ours (286/100,000 person‐years) but, with only 18 KS cases, considerably less precise (95% CI: 201–537/100,000 person‐years). In addition, having been conducted early in the ART rollout, the estimate from Asiimwe et al. may reflect an overall lower underlying CD4 count structure than our present population. Most recently, Akarolo‐Anthony et al. also used a record linkage algorithm to match HIV‐infected patients (presumably a mix of both those on and off ART) registered at healthcare facilities to the cancer registry in Abuja, Nigeria 29. Estimated KS incidence was substantially lower at 4.9/100,000 person‐years, over 90% less than estimates in comparable African populations. Besides the imperfect record linkage process, the authors suggest that the low incidence was because of the failure of clinicians to diagnose KS when it was present. We additionally speculate that the cancer registry did not record an important fraction of KS diagnoses made on clinical grounds only in ambulatory settings.

An approach more similar to ours was used to estimate KS incidence among HIV‐infected adults attending any one of six HIV clinics in Southern Africa IeDEA 13, 30 (Table 4). In this work, the incidence of KS off ART (1682/100,000 person‐years in South Africa from Bohlius et al. 13) was much higher than our estimate, but the two on‐ART estimates were lower (138/100,000 person‐years from South Africa 13 and 178/100,000 person‐years from 4 Southern African countries in Rohner et al. 30). Regarding the difference in off‐ART KS incidence, a difference in CD4+ T‐cell distribution between the regions is a theoretical explanation, but this is difficult to examine because Bohlius et al. provide CD4 count stratum‐specific KS incidences only according to CD4 count at clinic enrollment. Given this limitation, only the <50 cells/mm^3^ stratum can be appropriately evaluated, and when doing so, KS incidence in Southern Africa was still considerably higher (3281/100,000 person‐years) than our estimate (1818/100,000 person‐years). Another potential explanation is that although not described in the report, it is believed, on historical grounds, that a large fraction of the KS diagnoses in Southern Africa were clinical only, which are prone to false positivity 25. Regarding the on‐ART incidence estimate, examination of the CD4 count stratum‐specific estimates reveals that the overall higher estimate in East Africa is largely explained by our higher estimate in the <50 cells/mm^3^ group (1357/100,000 person‐years) compared to Bohlius et al. (188/100,000 person‐years) or Rohner et al. (403/100,000 person‐years). We speculate–as do the original authors–that this may be because of underascertainment of KS during the 2004 to 2007 period in Southern Africa. In fact, in Rohner et al., when analysis is restricted to 2007 to 2010, overall KS incidence on ART (224/100,000 person‐years) is much closer to our estimate, and, if children were excluded, it would even be closer. Finally, differences in underlying prevalence of KSHV may also contribute to the differences in KS incidence. Methodologic uncertainty in measuring KSHV infection status precludes clarity on this point, but we earlier found that, when using the same serologic assay in representative samples, KSHV prevalence was higher in Uganda than in Zimbabwe or South Africa 31.

As this study was conducted during the heart of the scale‐up of ART in East Africa, it offers insights into the impact of ART on the development of KS. In what we believe is a unique analysis, we found that the prevalence of KS declined at the largest of our sites (AMPATH). Among several potential explanations for this, the most obvious is that more HIV‐infected patients are achieving access to ART at earlier HIV disease stages and hence averting the development of KS while untreated in the community. KS incidence among those in care also declined, which is consistent with the growing proportion of patients on ART and achieving higher CD4 counts. Finally, the impact of ART is most clearly seen in the comparison of CD4 count stratum‐specific KS incidences between ART users and non‐ART users. Yet, none of these findings directly estimates what observers most commonly want to know: What is the reduction in KS incidence caused by ART if it could be tested in a randomized trial? As discussed in detail elsewhere 32, addressing this question is complex because of time‐dependent confounding/mediation by CD4+ T‐cell count in the available observational data. The solution to this analytical problem is more sophisticated than conventional regression allows and requires modeling techniques beyond the scope of the current work.

While we believe this work is among the most comprehensive attempts of KS incidence estimation in Africa by virtue of the provision of training and biopsy capacity to optimize the sensitivity and specificity of KS case ascertainment and the large well‐enumerated at‐risk denominator, there are still limitations. The first is that biopsy confirmation did not accompany all diagnoses, thus leaving open the possibility that some of the clinical‐only KS diagnoses were incorrect. We know that during this time period, at these sites, an important fraction of clinically suspected cases of KS concurrently sent for pathologic interpretation did not get confirmed as KS 25. Whether this fraction also holds true for clinical diagnoses not receiving pathologic interpretation is unknown. We speculate that the clinical training provided by IeDEA in KS differential diagnosis enhanced the positive predictive value of clinical‐only diagnosis, but this cannot be proven without performing a biopsy on all such instances. A second limitation is unlike a true research‐dedicated cohort, the patients in this study were not serially systematically evaluated for KS. Instead, KS ascertainment was made during the course of routine care by clinicians. We therefore may have missed cases and, more importantly, may have misclassified time of onset and hence grouping as prevalent versus incident KS or “off ART” versus “on ART.” Finally, some patients who developed KS while nominally on ART may have stopped using ART entirely and thus are misclassified in terms of ART exposure. While we do not believe that this is a sizeable fraction, it does require further investigation.

Our findings provide perspective about the impact of KS on the HIV healthcare system both now and into the future in East Africa. Our ASIR (334/100,000 person‐years when limited to adults and 247/100,000 when extrapolated to include children) provides a good estimate of the overall burden that KS poses. To provide context, the most common cancer among women in the United States is breast (ASIR = 125/100,000 person‐years) and among men is prostate (ASIR = 148/100,000 person‐years); the ASIR for all cancers combined is 460/100,000/person‐years 2. Although KS incidence is not of the same magnitude as the “Big 3” in Africa (HIV, tuberculosis, and malaria), its relevance in the context of cancer is clear. Regarding the future, the UNAIDS' 90‐90‐90 campaign 30 suggests that the ART‐treated HIV‐infected patient will become the rule rather than the exception in Africa. That said, our on‐ART KS incidence estimate foreshadows the future, and several aspects of this determination merit mention. First, although KS incidence on ART is considerably lower than off ART, it remains at levels that exceed the aforementioned most common cancers in general populations. Thus, ART has not eliminated KS. Second, incidence is dependent upon ambient CD4 count, and all discussions about KS incidence on ART must be conditioned on this important determinant. Among ART‐treated patients who had “normalized” their CD4 count (>500 cells/mm^3^), KS incidence was considerably lower (31/100,000 person‐years) but, again, still comparable to other important cancers (e.g., pancreas, stomach, and colon) as measured in resource‐rich regions 2. Therefore, it is now a research imperative to establish why HIV‐infected patients on ART still develop KS.

The same difficulties we described for the numerator and denominator in KS incidence estimation also apply to other cancers in the region, and hence, a healthcare system‐based approach to incidence estimation should be considered for these as well. This is most relevant for HIV‐infected populations for which the ART rollout has rapidly created a large well‐enumerated primary care system but will likely apply to HIV‐uninfected populations in the future as the lessons from the ART rollout get applied more generally. Again, the elegance of this approach rests primarily in the individual‐level enumeration of a large at‐risk population. If highly sensitive and specific diagnosis of a given cancer can be added to the population, accurate and precise cancer incidence estimation follows. We believe that this is most immediately plausible for cancers which feature feasible screening and case ascertainment, such as cervical, breast, and hepatocellular.

In summary, we have demonstrated how a strategic investment to improve diagnosis in combination with an already well‐enumerated large denominator allowed us to use a healthcare system‐based population to derive an improved estimate of the incidence of KS in East Africa. We found that, overall, KS incidence exceeds the most common cancers in resource‐rich settings, but it is declining as the ART rollout extends. Yet, KS incidence among patients on ART does not fall to zero, even among those with “normalized” CD4 counts. Attention now must turn to why HIV‐infected patients on ART still develop KS.
